# The everchanging Sky-Tower – an apparent giant

**DOI:** 10.1177/03010066231222526

**Published:** 2023-12-21

**Authors:** Dietrich S. Schwarzkopf, Annelise Kolf, Cathy Lai, Tina Huang, Shuji Kinoshita

**Affiliations:** University of Auckland, Aotearoa, New Zealand

**Keywords:** perception, scene perception, shapes/objects, spatial vision

## Abstract

For over a quarter-century, the Sky-Tower has dominated the skyline of Auckland Tāmaki Makaurau. Despite its imposing height, observers anecdotally report odd fluctuations in how big it appears. From certain angles, it can look positively stumpy. Such misperceptions can be bewildering and perilous when it happens whilst driving. Here, we characterise this strange illusion in the hopes of better understanding its cause.

Standing 328 m tall, the Sky-Tower should rise above all its neighbouring buildings^1^. The next tallest^2^ only measures 180 m. However, anecdotal reports suggest our perception of the Sky-Tower is not constant. Many newcomers to Auckland mention fluctuations in its apparent size. From some angles, especially iconic views of the skyline, it usually appears like a tall, thin needle. In contrast, from other locations it can seem oddly short and squat (Figure 1A-D).

**Figure 1.**
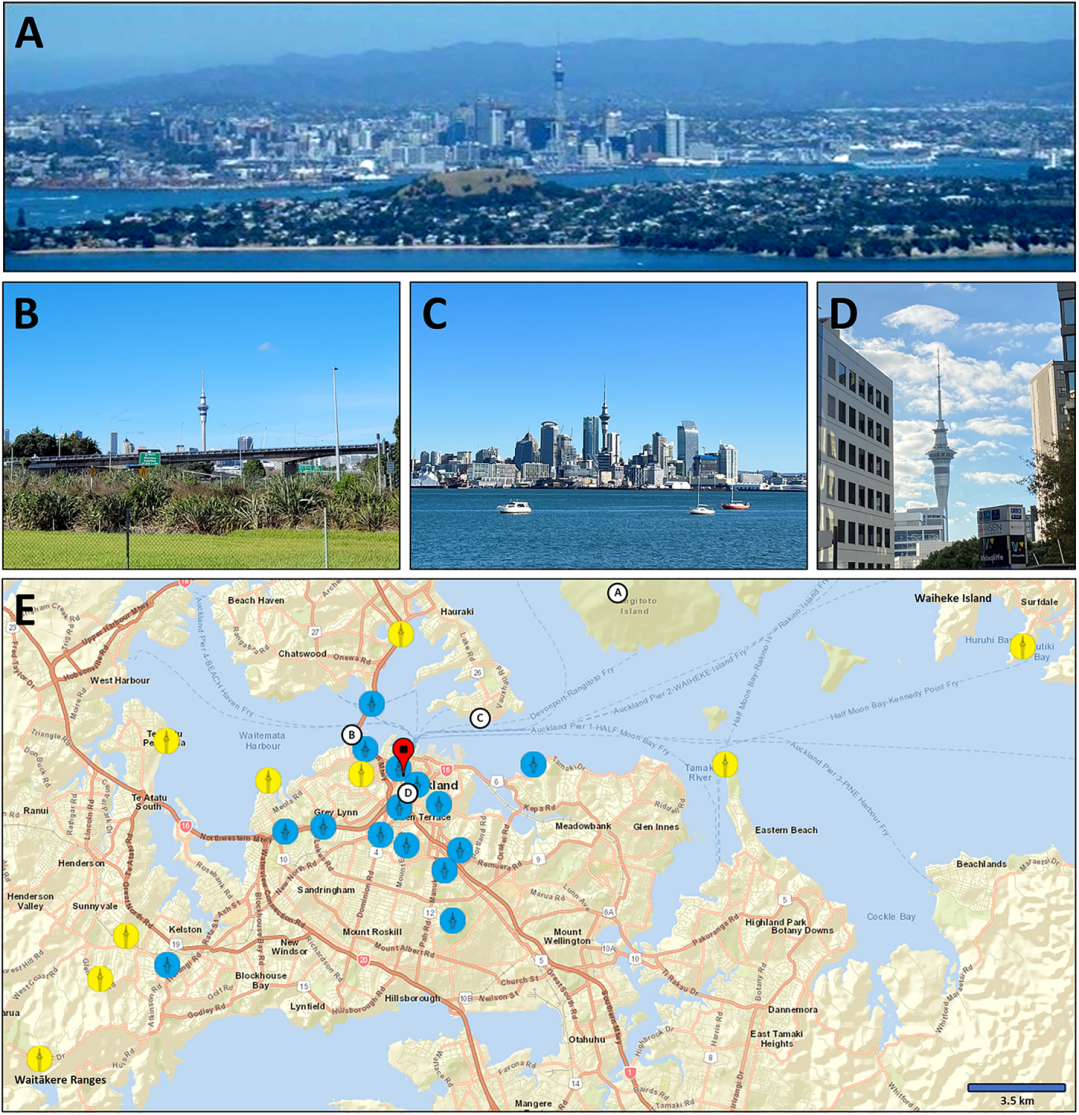
Photos poorly reflect the fluctuations in the apparent size of the Sky-Tower. (A) Yet, the reader may notice that in iconic views the Tower appears as an imposing feature of the skyline. (B) Viewed from further afield, it can appear as a tall singular spire. (C) It seems hardly taller than the surrounding high rises in other locations. (D) From up-close, the Tower often appears relatively short and squat. (E) DSS's judgements of the Tower's apparent width at representative locations across the city, seen either as wide or thin (as indicated by the icons). The dropped pin denotes the Sky-Tower's location. The white letter labels denote the locations from which the photographs in A-D were taken.

To get to the bottom of this mystery, author DSS judged his impression of the Tower's size from 36 vantage points across Auckland. He rated its appearance on three dimensions: (1) whether the Tower appeared tall or short, (2) thin or wide, and (3) by estimating how much taller it appeared relative to the next tallest building visible. The Tower's apparent width changed with distance: Viewed from the near the city centre, it appeared wide, but from afar, such as Waiheke Island or the Waitākere Ranges, it looked thin (Figure 1E). Similarly, it appeared tall from afar but short from up-close, although this relationship was less clear. Interestingly though, the closer he was to the Tower, the *taller* he judged it relative to its neighbours (Spearman's ρ = −0.53, *p* = .0009). In contrast, there was no significant relationship between his altitude and relative height judgements (ρ = −0.16, *p* = .3407).

Since these measurements are still anecdotal, DSS roped four Optometry Honours students into testing these relationships in a larger sample. We asked 23 observers (7 male, 16 female, aged 21–25 years, who all gave written informed consent) to make similar judgements. Instead of bussing all these observers around the city—something anyone familiar with Auckland's traffic situation will recognise as nigh impossible—we instead opted to present people with photographs. We used mobile phone cameras to take 62 geotagged pictures of the Sky-Tower from various locations, ensuring similar atmospheric and daylight conditions, always in landscape orientation and without zoom. Using http://testable.org, we then showed these images to observers on a computer screen in the psychophysics lab. They again judged how much taller the Sky-Tower appeared relative to the next tallest building in the picture, using a slider. They also made a binary choice of its shape being either “tall-and-slim” or “short-and-wide.” On average, participants tended to say the Sky-Tower photographed from afar was tall-and-slim, while from nearer locations it seemed short-and-wide (ρ = 0.69, *p* < .0001). However, in contrast to DSS's anecdotal reports, the judgement of the Tower's relative height did not anticorrelate with distance (ρ = 0.2, *p* = .1164). Interestingly, we also found a modest altitude effect: relative height was taller when seen from higher vantage points (ρ = 0.37, *p* = .0028). In contrast, binary judgement of the aspect ratio did not depend significantly on altitude (ρ = 0.13, *p* = .3047). The two perceptual measures themselves were strongly correlated (ρ = 0.55, *p* < .0001).

With their short focal lengths, phone cameras poorly capture the experience of the Sky-Tower seen in situ. Photos flatten the depth perspective. From beyond 10 km, the Sky-Tower is almost imperceptibly small in these photos compared to its surroundings. We therefore only used photos taken at smaller distances. The reduced dynamic range could explain why the lab experiment did not replicate the relationship DSS found between relative height judgements and distance. Conversely, the strong size impression in the three-dimensional (3D) real-world setting could mask the more subtle association between altitude and relative height judgements we found in the lab.

Nonetheless, our findings suggest that the Sky-Tower's apparent height or squatness depends on distance, confirming our outgoing suspicions about this illusion. The effect could be related to phenomena like the Moon Illusion, whereby the moon appears larger when near the horizon than up in the sky. This may reflect misapprehensions of how far away the object is (Gregory, 2008). A similar mechanism could, therefore, affect our judgement of other distant tall buildings. We are unaware of similarly striking perceptual fluctuations, neither about comparable landmarks like Toronto's CN Tower, Seattle's Space Needle, or the Eiffel Tower, nor about skyscrapers like the Burj Khalifa or the Shanghai Tower. Some visitors of London, UK, anecdotally report that the clock tower housing Big Ben appears weirdly squat when viewed up-close in Westminster. This effect, however, still feels modest compared to what our perception does to the Sky-Tower. The phenomenon bears some similarity with the vista paradox in which tall towers can appear to perceptually shrink on approach when viewed through a window or framed by a corridor or narrow street (Costa & Bonetti, 2017; Todorovic, 2016; Walker et al., 1989). In contrast, the apparent size of the Sky-Tower varies substantially between locations in the city but not over a short approach of only a few meters within the *same* location, as in the vista paradox.

At shorter viewing distances, Auckland's terrain and architecture might sometimes block the view of the thinner stem, such that the “bulb” near the top seems to hover only barely above neighbouring rooftops. However, this alone is insufficient as an explanation; one can experience its squatness from unimpeded views. Curiously, from some views no surrounding buildings are visible at all, while from other directions the Tower stands amidst a cluster of high rises. Perhaps against the backdrop of Rangitoto, the surrounding architecture can blend into the background.

A famous children's book by Michael Ende describes a *Scheinriese*, an apparent giant, who appears enormous from a distance but is like a typical human up-close (Ende, 1960). It would appear the Sky-Tower replicates this puzzling breakdown of size constancy in the heart of our city. Future investigations could survey passers-by at multiple vantage points. Alternatively, one might reproduce the perceptual distortion using a 3D virtual reality environment. This would have the added benefit of allowing manipulation of the Tower's size enabling careful psychophysical measurements.

## Notes

1.
https://www.skycityauckland.co.nz/sky-tower

2.
https://www.skyscrapercenter.com/city/auckland

## References

[bibr1-03010066231222526] Costa M. Bonetti L. (2017). Linear perspective and framing in the vista paradox. Perception, 46(11), 1245–1268. 10.1177/0301006617713091 28649908

[bibr2-03010066231222526] Ende M. (1960). Jim Knopf und Lukas der Lokomotivführer. Thienemann.

[bibr3-03010066231222526] Gregory R. L. (2008). Emmert’s law and the moon illusion. Spatial Vision, 21(3–5), 407–420. 10.1163/156856808784532509 18534112

[bibr4-03010066231222526] Todorovic D. (2016). A real-life size perception paradox. Journal of Vision, 16(12), 297. 10.1167/16.12.297

[bibr5-03010066231222526] Walker J. T. Rupich R. C. Powell J. L. (1989). The vista paradox: A natural visual illusion. Perception & Psychophysics, 45(1), 43–48. 10.3758/bf03208031 2913569

